# Curiosity in childhood and adolescence — what can we learn from the brain

**DOI:** 10.1016/j.cobeha.2021.03.031

**Published:** 2021-06

**Authors:** Matthias J Gruber, Yana Fandakova

**Affiliations:** 1 Cardiff University Brain Research Imaging Centre (CUBRIC), School of Psychology, Cardiff University, United Kingdom; 2 Center for Lifespan Psychology, Max Planck Institute for Human Development, Berlin, Germany

## Abstract

Accumulating evidence in adults has shown that curiosity and surprise enhance memory via activity in the hippocampus, prefrontal cortex, and dopaminergic areas. Based on findings of how these brain areas and their inter-connections develop during childhood and adolescence, we discuss how the effects of curiosity and surprise on memory may develop during childhood and adolescence. We predict that the maturation of brain areas potentially related to curiosity elicitation (hippocampus, anterior cingulate cortex [ACC], prefrontal cortex) and protracted development of hippocampal-PFC and ACC-PFC connectivity lead to differential effects of curiosity and surprise on memory during childhood and adolescence. Our predictions are centred within the PACE (Prediction-Appraisal-Curiosity-Exploration) Framework which proposes multiple levels of analyses of how curiosity is elicited and enhances memory.

## Highlights

• Curiosity enhances memory via the hippocampus, prefrontal cortex, and ventral striatum.
• Development of curiosity and its effect on memory in childhood/adolescence not well understood.
• Maturation of curiosity-promoting brain functions might contribute to increasing benefits of curiosity for learning.
• Harnessing curiosity in education might need differential approaches across child development.

**Current Opinion in Behavioral Sciences** 2021, **39**:178–184

This review comes from a themed issue on **Positive Affect**

Edited by **Henk van Steenbergen**, **Disa Sauter**, **Blair Saunders** and **Gilles Pourtois**

For a complete overview see the Issue and the Editorial

Available online 1st May 2021

**https://doi.org/10.1016/j.cobeha.2021.03.031**

2352-1546/© 2021 The Authors. Published by Elsevier Ltd. This is an open access article under the CC BY license (http://creativecommons.org/licenses/by/4.0/).

## Introduction

Curiosity, the desire to acquire new information, is often described as an epistemic emotion and is accompanied by positive affect [1]. It has been shown to be a powerful driver of learning, especially in children [2]. In educational settings, curiosity for scientific knowledge is a major motivation for long-term involvement in STEM subjects and predicts academic performance [3,4]. Experiencing and expressing higher curiosity during kindergarten predicts academic achievement in primary school, with an even larger influence in children from families with lower socio-economic status [5^•^]. But what are the neural underpinnings underlying the positive effects of curiosity on learning and memory, and how do they develop? Answers to this question would ultimately allow us to design tailored educational approaches to optimally harness how curiosity differently affects learning across development. Furthermore, a neuroscientific approach to study curiosity development offers a unique opportunity to investigate how neural mechanisms underlying learning are modulated by the drive to learn and the satisfaction that comes from learning the desired information.

**Figure 1:**
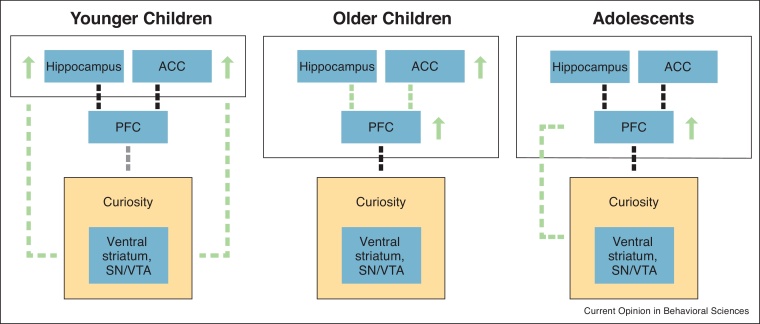
Predictions on the neural correlates of curiosity elicitation during development. Our predictions are centred within the PACE (Prediction, Appraisal, Curiosity, and Exploration) Framework which proposes multiple levels of analyses of how curiosity is elicited and how it enhances memory [21^••^]. In the PACE Framework, curiosity is triggered by significant prediction errors in the hippocampus and the anterior cingulate cortex (ACC). While prediction errors in the hippocampus are proposed to generally result from encountering novel or unexpected contexts (i.e. contextual prediction errors), prediction errors in the ACC are triggered by cognitive conflict resulting from previous knowledge (i.e. informational prediction errors). PACE suggests that these prediction errors are appraised via lateral prefrontal cortex (PFC) mechanisms in order to evaluate whether the information could be valuable in the future. When curiosity is triggered, a PACE cycle enhances memory encoding through increased attention, exploration, and information-seeking via the dopaminergic circuit, leading to enhanced hippocampus-dependent memory of curiosity-related information. We predict that in young children (left graph) due to ongoing development of the hippocampus and the ACC (indicated by green arrows), hippocampus-dependent and ACC-dependent prediction errors will elicit curiosity in a rather obligatory manner without a strong contribution of PFC-related appraisal processes (indicated by green dashed lines). In older children (middle graph), we expect that age differences in curiosity will primarily result from ongoing development of ACC, PFC along with hippocampal-PFC and ACC-PFC connections (indicated by green arrows and dashed lines, respectively). In adolescents (right graph), protracted PFC development along with connections between the PFC and dopaminergic circuit areas (i.e. ventral striatum and SN/VTA) are proposed as the key mechanisms eliciting curiosity. While brain development in these age groups is not limited to the highlighted regions and their connections, we only depicted those aspects that are proposed to drive corresponding differences in curiosity.

A plethora of research has consistently demonstrated that infants and young children explore their environment actively in systematic ways, driven by a drive to reduce uncertainty and to close knowledge gaps — both key markers of curiosity [6, 7, 8,9^•^]. In addition, the educational literature has emphasized the cognitive and affective mechanisms promoting school-aged children’s and adolescents’ long-lasting interest and curiosity in such domains as mathematics or physics [10, 11, 12]. Yet, we have a limited understanding of how different levels of curiosity affect children’s learning because hardly any studies to date have directly measured curiosity or asked children to report on their states of curiosity. Thus, children’s (subjective) desire to learn and satisfaction in experiencing desired information has rarely been taken into account when examining curiosity-based learning. However, a fledgling line of research in psychology and neuroscience on curiosity in young adults (i.e. 18–30 years of age) has consistently demonstrated how pre-information curiosity, post-information interest, and surprise enhance learning and memory in adults [13, 14, 15, 16, 17, 18]. These studies have been employing a trivia paradigm in which participants anticipate answers to general knowledge questions that are associated with varying levels of curiosity about the answer. Using an age-appropriate version of the trivia paradigm, we recently investigated how curiosity and surprise affect memory in children between 10 and 14 years [19^•^]. We found that younger children (10−12 years) and adolescents (12–14 years) demonstrated enhanced memory for answers to trivia questions for which they were curious relative to answers to trivia questions about which they were not curious. Furthermore, we found that adolescents — but not children — showed better memory for answers to trivia questions that they judged as more interesting than initially expected. These initial results suggest that states of curiosity can indeed be harnessed to facilitate learning in children and adolescents. However, they also point to potential differences in the underlying mechanisms of how positive surprise affects learning across development (Figure 1).

To better understand how curiosity-based learning might develop, we turn to theoretical ideas and current findings in cognitive neuroscience for this opinion piece. In adults, cognitive neuroscience research has started to differentiate the components and neural circuits associated with curiosity-based learning, thereby bridging the fields of memory and motivation [14,15,20,21^••^,^22^]. Of note, these two rich fields have mostly been studied in isolation, especially in children. We aim to close this gap by integrating recent findings and theoretical ideas on the neural mechanisms of curiosity with findings from developmental cognitive neuroscience to identify candidate mechanisms facilitating the differential effects of curiosity and interest on learning and memory across development.

## The Prediction, Appraisal, Curiosity, and Exploration (PACE) Framework and its relationship to child and adolescent development

An emerging field in neuroscience on curiosity has started to elucidate the neural underpinnings underlying states of curiosity in hon-human primates as well as humans (for reviews, see Refs. [13,21^••^,23^••^,^24^,^25^]. Across different experimental manipulations of curiosity (e.g. trivia questions, magic tricks, blurred images, or morbid stimuli), studies in humans have consistently shown that states of curiosity elicit activity in dopaminergic circuit regions, specifically in the ventral striatum [14,15,20,22,26,27,28^••^]. In addition, one study has shown that the enhancing effects of curiosity on human long-term memory are supported by activity in the ventral striatum and hippocampus suggesting enhanced hippocampus-dependent memory formation via interactions with the dopaminergic circuit [15] (see also, [22]).

Recently, Gruber and Ranganath proposed a framework that integrates the emergent research on curiosity, drawing from a broad range of evidence and theoretical models from psychology and neuroscience on how novelty and prediction errors trigger exploration and information-seeking [21^••^]. Specifically, Gruber and Ranganath proposed that the effects of curiosity on memory can be understood as emerging from a cycle that involves Prediction errors, Appraisal, Curiosity, and Exploration (PACE). According to this framework, curiosity is first triggered by significant prediction errors, in particular hippocampus-dependent contextual prediction errors and anterior cingulate cortex-dependent informational prediction errors. While prediction errors in the hippocampus are proposed to generally result from encountering novel or unexpected contexts, prediction errors in the anterior cingulate cortex (ACC) depend on cognitive conflict resulting from previous knowledge. PACE suggests that these prediction errors are appraised via lateral prefrontal cortex (PFC) mechanisms as an indicator of information that could be valuable in the future. This cycle enhances memory encoding through increased attention, exploration, and information-seeking via the dopaminergic circuit and enhances hippocampus-dependent memory of curiosity-related information [21^••^]. Below, we outline how the proposed processes within the PACE framework might help to ultimately better understand the development of curiosity and its effect on memory (Figure 1).

### Age differences in context-based and information-based prediction errors

#### Hippocampal context-based prediction errors

It has been proposed that the hippocampus forms cognitive maps that allow one to generate predictions based on past experiences with similar contexts and situations [29]. Violations of such generated predictions, in turn, may lead to hippocampal responses that can potentially trigger exploration to resolve this uncertainty and to refine cognitive maps [29]. Thus, the hippocampus can be seen as providing the foundation for curiosity through novelty-based or context-based prediction errors that lead to an inherent drive for curiosity-stimulated exploration [21^••^]. Consistent with findings on how the hippocampus supports exploratory eye movements related to prediction errors and novelty [30, 31, 32], it has been shown that eye movements related to curiosity predict exploration and attention towards novel information [33,34]. Furthermore, one study investigated individual differences in the strength of one major anatomical pathway connecting the hippocampus with the PFC — the fornix — and its relationship with curiosity [35]. The authors found that individual differences in the microstructure of the fornix predicted specifically diversive curiosity — a curiosity trait that is related to broad exploration triggered by novel events [35,36].

Consistent with these findings in young adults, infants show visual preferences for exploring novel objects, and young children prefer to explore objects if they do not have complete understanding of their functioning [37,38]. At the same time, the hippocampus continues to develop in early and middle childhood and supports improvements in memory precision and flexibility [39]. Continued hippocampal maturation may thus contribute to age differences in the ways in which context-based prediction errors stimulate curiosity in younger children. In addition, studies in young adults have shown that surprising information elicits functional connectivity between the hippocampus and the PFC [40, 41, 42]. Critically, there are developmental differences in connectivity between the hippocampus and the PFC (e.g. via the fornix or the uncinate fasciculus), which may also contribute to age differences in how hippocampus-mediated prediction errors elicit curiosity. The uncinate fasciculus continues to develop throughout middle childhood [43] and the strength of the uncinate fasciculus microstructure correlates with age-related increases in the ability to modulate attention towards relevant information in children (7–11 years) [44]. Furthermore, a longitudinal study [45] using resting-state functional magnetic resonance imaging found that hippocampus-PFC functional connections only emerged by 13 years of age, suggesting that the transition to adolescence may be an important period for the development of the connections between the hippocampus and the PFC.

Therefore, hippocampal context-based prediction errors may support the computation of unexpected or novel contextual information which may provide the foundation for curiosity in childhood and adolescence. This process may differ from that observed in adults because the relevant functions of the hippocampus and its connections to other subcortical and cortical networks are still developing. While we expect that hippocampal maturation would represent the major source of age differences in hippocampal prediction errors earlier in childhood, changes in hippocampal-PFC connections in the transition to adolescence are expected to make greater contributions to curiosity and its effect on memory later in child development.

#### Information-related prediction errors in the ACC

While the hippocampus might compute contrasts in map-like representations elicited by prediction errors, PACE further proposes that the ACC supports the cognitive conflict that is experienced due to information-based prediction errors and information gaps [21^••^]. This idea is in line with the theoretical conceptualization of information gaps in terms of cognitive conflict [46] and the neuroscientific literature that has shown enhanced ACC and lateral PFC activity when participants experience cognitive conflict (e.g. Refs. [47,48]), including increased ACC activity during the tip-of-the-tongue experience — a phenomenon that has been related to high levels of curiosity [49,50]. Consistent with the proposed ACC-related cognitive conflict component within the PACE framework, several studies have shown involvement of the ACC when humans and non-human primates await or choose information associated with high curiosity, potentially supporting the idea that the ACC might signal information gaps due to cognitive conflicts that can trigger curiosity [15,22,26,27,28^••^,^51^].

During development, age differences in information-related prediction errors supported by the ACC may contribute to differences in whether and how such prediction errors stimulate curiosity. While some signatures of cognitive conflict are present even in infancy [52], the ACC continues to mature in childhood and adolescence [53]. In particular, the amplitude of the error-related negativity (ERN), an EEG component associated with the detection and processing of cognitive conflict, increases with age between 8 and 19 years [54]. Thus, protracted development in the neural circuits supporting conflict processing could alter how information-related prediction errors affect the stimulation of curiosity during development. In particular, ACC input associated with conflict monitoring may play an important role in stimulating appraisal-based processes in the lateral PFC. For example, Fandakova and colleagues [55^•^] found that 8−12 year old children engaged the ACC and anterior insula more strongly during inaccurate and uncertain memory responses, but only 10–12-year-olds recruited the lateral PFC more strongly for decisions to report uncertainty. Further longitudinal analyses demonstrated that 8−10-year-olds who exhibited greater activation of regions associated with cognitive conflict at a first assessment showed greater increases in PFC activation for uncertain responses 1.5 years later [55^•^]. Consistent with findings of protracted network segregation in childhood [56, 57, 58], this initial evidence might suggest that input signals from regions associated with cognitive conflict might contribute to the development of more differentiated appraisal in PFC that would ultimately lead to curiosity. Thus, one hypothesis for future research is that experiencing more information-related prediction errors in a given domain earlier in childhood may contribute to faster development of more efficient PFC-based appraisal in the service of curiosity.

Taken together, after experiencing information gaps due to cognitive conflicts, children and adolescents may become more likely to engage in more differentiated curiosity-driven exploration with increasing age as ACC-based conflict processing improves and contributes to the development of more efficient and differentiated PFC-based appraisal.

### Protracted development of appraisal supported by the lateral PFC

The PACE framework lays out that context-based and information-based prediction errors do not elicit curiosity in an obligatory manner, but that prediction errors are appraised involving lateral PFC functions [21^••^] (see also, Refs. [59,60]). According to PACE, appraisal of prediction errors can lead to different degrees of curiosity or alternatively to anxiety-related inhibition if one does not have sufficient capability to resolve the uncertainty [21^••^,^61^]. Consistent with the idea of prefrontal appraisal processes, several neuroimaging studies in young adults have shown lateral PFC activity along with activity in dopaminergic mesolimbic regions when curiosity is elicited [14,15,27,28^••^] (for reviews, see Refs. [21^••^,23^••^]). These findings suggest that PFC-based appraisal may be needed to stimulate dopaminergic functions to modulate hippocampus-dependent learning.

Lateral PFC is among the brain regions that shows protracted maturation up to young adulthood [62, 63, 64]. Gray matter volume in lateral PFC increases in early childhood, followed by thinning starting around age 9–10 years and continuing through adolescence [65]. A recent study provided hints that structural changes in the PFC are related to the development of appraisal processes [66]. This longitudinal study examined metamemory development in children between 7 and 15 years. Metamemory — the ability to appraise, self-reflect, and regulate learning and memory outcomes — continued to improve over time aligned to structural changes in the PFC. These findings based on the appraisal of memory retrieval suggest that appraisal processes may develop throughout adolescence, reflecting protracted PFC maturation (see also Ref. [55^•^]). Thus, across development curiosity may be elicited to a different degree based on the maturational status of lateral PFC. More specifically, if appraisal processes are still developing in younger children, we expect that (1) they show less lateral PFC modulation by context-based and/or information-based prediction errors and (2) are overall more likely to report higher curiosity rather than differentiating between information associated with high versus low curiosity as older children and adults do. Evolutionarily, there might be an obligatory drive for curiosity in early development or at least an inherent bias towards curiosity over anxiety (*cf.* Ref. [29]) as prefrontal appraisal processes are still maturing. The protracted development of appraisal processes aligned to PFC maturation may be one neural mechanism enabling an extended exploratory childhood period [67^••^], in which context-related and information-related prediction errors may trigger curiosity directly. Future research is necessary to test these hypotheses, but they are consistent with observations that younger children are more likely to show greater interest across a variety of different academic domains, whereas older children have fewer, but clearly differentiated domains of interest [68]. On the neural level, our hypotheses are consistent with research demonstrating that the extent to which lateral PFC activity selectively supports task-relevant versus task-irrelevant information increases with age in 8–13 year-olds [44].

Taking a neuroscientific approach to the development of appraisal processes in service of curiosity and learning can offer unique insights into the interactions of processes associated with the drive to learn (related to dopaminergic circuit functions) and learning itself (related to the hippocampus and memory circuits more generally). For example, our findings that showed that information prediction errors enhanced memory in adolescents more strongly than in children [19^•^] point to an interaction between the developing PFC appraisal processes and dopaminergic neuromodulation of hippocampus-dependent memory. These interactions are particularly prominent in guiding learning in adolescence [69, 70, 71] and may enhance curiosity-based learning. In younger children in contrast, the satisfaction associated with learning may emerge from direct triggering of curiosity by context-related and information-related prediction errors. These ideas are consistent with postulated changes in the extent to which cognitive and affective components (*cf.* Ref. [68,72]) drive curiosity-based learning in development. Ultimately, a neuroscientific approach to the development of appraisal processes in curiosity-based learning will offer unique insights into how these processes interact with the drive to learn within the dopaminergic system and hippocampus-based learning and exploration. In addition, future research might eventually point to optimal ways to harness curiosity-based learning across child development.

## Conclusion

The PACE framework offers an excellent starting point for investigating how brain maturation contributes to curiosity and its effects on learning in childhood and adolescence. First, we expect that hippocampus-related and ACC-related prediction errors (i.e. via novelty and information gaps, respectively) and their effects on curiosity-driven exploration underlie age differences during development due to the ongoing development of these structures. Second, based on the different maturational trajectories of the hippocampus and the ACC, we propose that younger children will show differences to adults in hippocampus-related novelty prediction errors and how they stimulate curiosity. In contrast, older children and adolescents are expected to show differences to adults primarily in ACC-related prediction errors due to cognitive conflict. Finally, as the lateral PFC and its connections to hippocampus and ACC continue to develop, we expect more refined PFC-based appraisal for different strengths of prediction errors which parallels the development of more differentiated curiosity profiles on the behavioral level.

## Conflict of interest statement

Nothing declared.

## References and recommended reading

Papers of particular interest, published within the period of review, have been highlighted as:

• of special interest
•• of outstanding interest
